# Supplementary material for: A homozygous missense variant in CACNB4 encoding the auxiliary calcium channel beta4 subunit causes a severe neurodevelopmental disorder and impairs channel and non-channel functions
Source: PLoS Genet. 2020 Mar 16;16(3):e1008625. doi: 10.1371/journal.pgen.1008625 (PMC7176149; doi:10.1371/journal.pgen.1008625)
Supplement: S1 Text — (DOCX) [file pgen.1008625.s001.docx]

**Supporting Information:**

**S1 Text**

**Clinical report of the patients**

The two siblings, a girl and a boy, were the only children of healthy, consanguineous parents of Turkish origin. Pregnancy and birth were uneventful in both children; they were born at term with normal measurements.

The boy (patient 1) came to our attention because of severe delay in global development and absence of eye contact within the first weeks of life. He developed focal seizures at the age of 3 years which well responded to antiepileptic medications. He was seizure-free for several years under low-dose antiepileptic treatment with valproic acid. At last examination he was 15 years old and was able to roll over, but had not acquired any other developmental milestones or speech. He showed severe truncal muscular hypotonia and dystonic and athetoid movements. In addition, he was close to blind, visual evoked potentials were absent in repeated measurements. Hearing was normal, he well responded to sounds and speech. All measurements were below the third centile with OFC of -2.1z, length of -3.8z and weight of -3.7z. A first brain MRI at the age of 3 years and a second at the age of 14 years demonstrated severe cerebellar atrophy in both and mild ventricular enlargement in the second one. Ophthalmologic examination at age 15 years did not reveal anomalies, in particular no retinal changes and no optic atrophy.

The older sister (patient 2) showed a very similar phenotype. She came to attention within the first months of life due to the absence of motor development and eye contact. At the age of 6 months, she developed tonic and later focal to bilateral tonic-clonic seizures, but her seizures did not respond to multiple antiepileptic drug therapy and vagus nerve stimulation. Over the next years she did not develop any motor or language skills or visual abilities. At last examination she was 22 years old and wheelchair-bound. She was a severely disabled, friendly woman with severe truncal hypotonia and dystonic-athetoid cerebral palsy. She was blind but she well responded to speech and sounds. Her parents reported daily tonic-clonic seizures when she gets awake and grand mal seizures twice a month. Weight and length were below the third centile (-4.1z and -2.7z, respectively), the OFC was close to normal (-1.9z). Electroencephalogram revealed multiregional sharp waves, structural deficiency and slowed background activity. Visual evoked potentials were reduced in amplitudes. Otherwise, the ophthalmologic examination gave normal results; in particular, the retina was normal. Brain MRI at the age of 6 months was normal but showed a severe cerebellar atrophy at the age of 8 years.
