# Supplementary figures and images for: A homozygous missense variant in CACNB4 encoding the auxiliary calcium channel beta4 subunit causes a severe neurodevelopmental disorder and impairs channel and non-channel functions
Source: PLoS Genet. 2020 Mar 16;16(3):e1008625. doi: 10.1371/journal.pgen.1008625 (PMC7176149; doi:10.1371/journal.pgen.1008625)

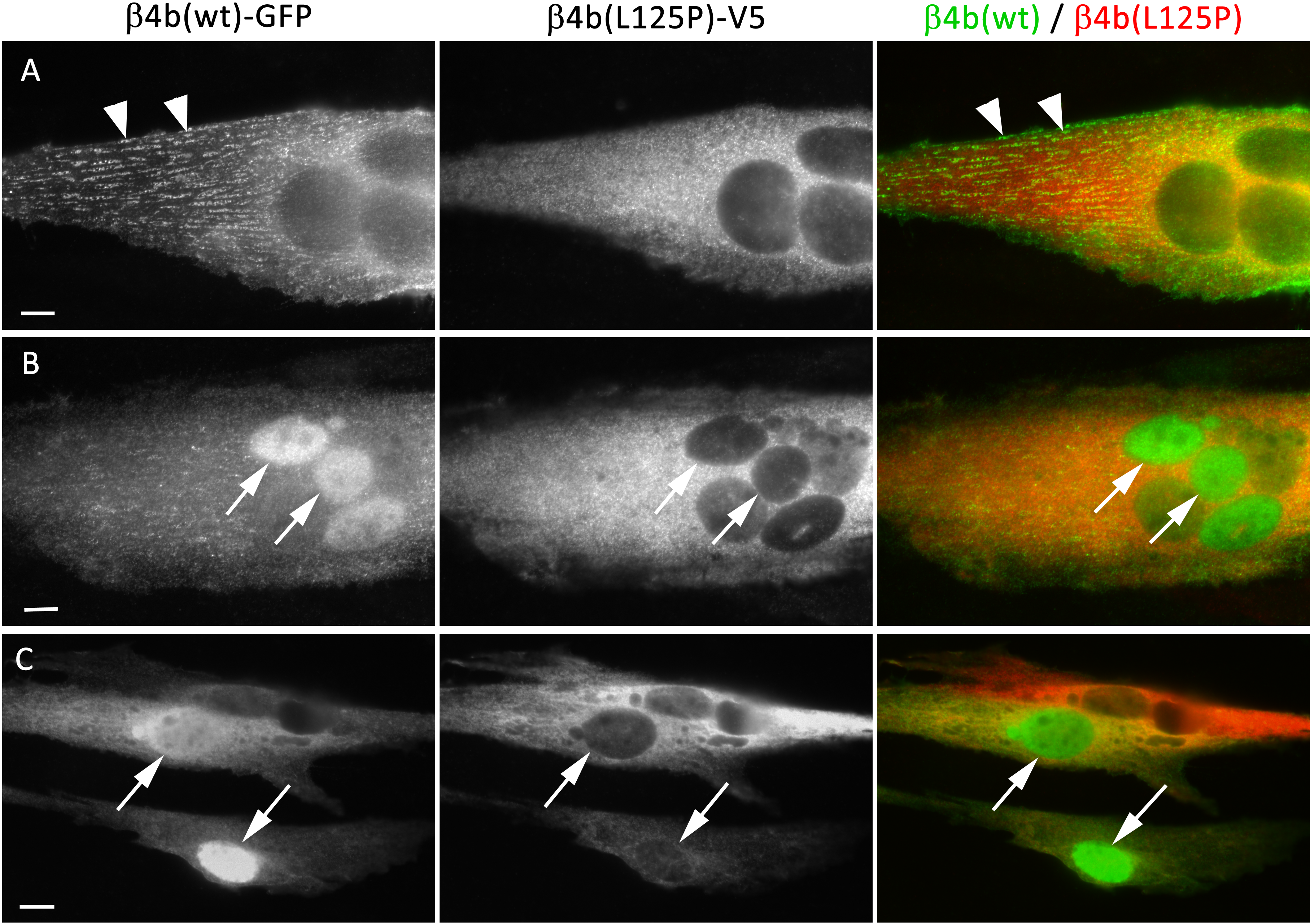

Supplement: S1 Fig — Dysgenic (CaV1.1-null) muscle cells were transfected with CaV1.2 together with both β4b(wt)-GFP plus β4b-L125P-V5 to mimic the situation in heterozygous carriers of the CACNB4 variant, and immunolabeled with anti-GFP (green) and anti-V5 (red). (A) The β4b(wt)-GFP displayed the typical clustered distribution, resembling the β4b subunit incorporated into calcium channel complexes (examples indicated by arrow heads). In contrast, the co-expressed mutant β4b-L125P-V5 was evenly dispersed throughout the cytoplasm, indicating its failure to associate with the pore-forming CaV1.2 subunit. (B and C) In quiescent cells (immature myotubes (B) or myoblasts (C)) that displayed nuclear targeting of the wildtype β4b(wt)-GFP subunit, the mutant β4b-L125P-V5 failed to accumulate in the nuclei (examples indicated by arrows). N = 5. Scale bars, 10 μm. (TIF) [file pgen.1008625.s002.tif]
