# Supplementary material for: A homozygous missense variant in CACNB4 encoding the auxiliary calcium channel beta4 subunit causes a severe neurodevelopmental disorder and impairs channel and non-channel functions
Source: PLoS Genet. 2020 Mar 16;16(3):e1008625. doi: 10.1371/journal.pgen.1008625 (PMC7176149; doi:10.1371/journal.pgen.1008625)
Supplement: S1 Table — Trio-exome data were filtered for potentially pathogenic de novo variants absent in the general population (dbSNP138, 100 Genomes Project, Exome Variant Server, ExAC Browser, and gnomAD Browser) and rare biallelic variants with minor allele frequency (MAF) <0.1% and no homozygous carriers in the aforementioned databases. MetaDome web server (https://stuart.radboudumc.nl/metadome) combines resources and information from genomics and proteomics to improve variant interpretation by transposing this variation to homologous protein domains. It visualizes meta-domain information and gene-wide profiles of genetic tolerance [70]. The constraint score shown in gnomAD is the ratio of the observed/expected (o/e) number of missense variants in that gene. The functional impact of the identified variants was predicted by the Combined Annotation Dependent Depletion (CADD) tool, the Rare Exome Variant Ensemble Learner (REVEL) scoring system, and the Mendelian Clinically Applicable Pathogenicity (M-CAP) Score. CADD is a framework that integrates multiple annotations in one metric by contrasting variants that survived natural selection with simulated mutations. Reported CADD scores are phred-like rank scores based on the rank of that variant’s score among all possible single nucleotide variants of hg19, with 10 corresponding to the top 10%, 20 at the top 1%, and 30 at the top 0.1%. The larger the score the more likely the variant has deleterious effects; the score range observed here is strongly supportive of pathogenicity, with all observed variants ranking above ~99% of all variants in a typical genome and scoring similarly to variants reported in ClinVar as pathogenic (~85% of which score >15) [96]. REVEL is an ensemble method predicting the pathogenicity of missense variants with a strength for distinguishing pathogenic from rare neutral variants with a score ranging from 0–1. The higher the score the more likely the variant is pathogenic [97]. M-CAP is a classifier for rare miss [file pgen.1008625.s003.pdf]

S1 Table

Chr.	Genomic position	Gene	mRNA reference number	Nucleotide change	Amino acid alteration	MetaDome	gnomAD browser: MAF [%]	gnomAD o/e score	CADD (>20)	REVEL (>0.6)	M-CAP (>0.025)	OMIM phenotype and MIM number
**2**	27,305,013-27,305,033	*EMILIN1*	NM_007046.3	c.582_602del	p.(Arg196_Leu202del)	From neutral to slightly intolerant	0	0.86	19.43	–	–	–
**2**	33,413,803	*LTBP1*	NM_206943.2	c.1586A>G	p.(Gln529Arg)	Intolerant	0	0.98	24.2	0.419	0.039	–
**2**	152,737,327	*CACNB4*	NM_000726.3	c.377T>C	p.(Leu126Pro)	Highly intolerant	0	0.55	29.8	0.948	0.457	EA5; 613855 EIG9, EJM6; 607682
**15**	79,760,539	*MINAR1*	NM_015206.2	c.2564C>A	p.(Ser855Tyr)	Intolerant	0	1.05	25.7	0.502	0.054	–
**16**	72,133,108	*DHX38*	NM_014003.3	c.889C>T	p.(Arg297Cys)	Tolerant	0.001618	0.73	23.2	0.069	0.024	RP84; 618220
**17**	18,042,200	*MYO15A*	NM_016239.3	c.5083C>A	p.(Pro1695Thr)	Slightly tolerant	0.001213	0.96	24.3	0.585	0.166	DFNB3; 600316
