# Supplementary material for: A homozygous missense variant in CACNB4 encoding the auxiliary calcium channel beta4 subunit causes a severe neurodevelopmental disorder and impairs channel and non-channel functions
Source: PLoS Genet. 2020 Mar 16;16(3):e1008625. doi: 10.1371/journal.pgen.1008625 (PMC7176149; doi:10.1371/journal.pgen.1008625)
Supplement: S2 Table — Data are expressed as mean value ± SEM. (PDF) [file pgen.1008625.s004.pdf]

S2 Table

	V_{half} (mV)	n	I_{Peak} (pA)	n	I_{200ms}/I_{Peak}	n	G_{max} (nS/pF)	n	K_{act} (mV)	n
β4b	4.84 ±0.63	18	-36.79 ±7.25	18	0.78 ±0.02	18	0.72 ±0.15	18	3.49 ±0.14	18
β4b-L125P	5.23 ±0.58	18	-35.23 ±6.38	18	0.64 ±0.03	18	0.73 ±0.14	18	4.25 ±0.31	18
∅β	6.67 ±2.50	3	-3.32 ±1.15	10	0.48 ±0.09	3	0.14 ±0.05	3	4.71 ±0.23	3
