# Supplementary material for: A homozygous missense variant in CACNB4 encoding the auxiliary calcium channel beta4 subunit causes a severe neurodevelopmental disorder and impairs channel and non-channel functions
Source: PLoS Genet. 2020 Mar 16;16(3):e1008625. doi: 10.1371/journal.pgen.1008625 (PMC7176149; doi:10.1371/journal.pgen.1008625)
Supplement: Attachment — Submitted filename: PloS Genetics_reviewers comments.docx [file pgen.1008625.s005.docx]

The manuscript by Coste de Bagneaux and colleagues examines the functional impacts of a homozygous missense mutation in the *CACNB4* gene encoding the β4 subunit. Patients - two siblings of first-degree consanguineous healthy parents, carrying the L126P β4 mutation exhibit severe neurodevelopmental disorder, including intellectual disability and other comorbidities. The unique clinical data directly motivate the study. The results suggest that the mutation impairs presynaptic Cav2.1 channel function and disrupts nuclear targeting of β4. The paper is well-written and, in most cases, the conclusions are justified by results. Nuclear targeting of β4 is convincingly demonstrated. However, the suggested TNIK-β4 complex formation should be softened in the abstract.

In neurons, the auxiliary β4 subunit is associated with the pore-forming Cav2.1 (α-) subunit and modulates neurotransmission; β4 can also function as a chaperon, facilitating Cav2.1 channel complex formation and regulating surface expression. Unlike other β subunits, β4 can specifically be targeted to the nuclei of neurons to partner proteins involved in transcription and cell proliferation. At the molecular level, β4 interacts with the AID domain of the α-subunit, which binds to a hydrophobic groove of the β4’s guanylate kinase (GK) domain. Mutations in the pore-forming Cav2.1 or the auxiliary subunits are often associated with neurological disease; specifically, mutations in β4 can be implicated in epilepsy and ataxia in humans. The L126P residue-change in the GK domain likely impairs the intramolecular interaction of GK with the tandem srk homology (SH3) module of β4.

***Major comments***

1) The in silico pathogenicity prediction methods provided high scores for the L126P β4 and suggested this mutation was responsible for the neurological disease in the patients. Interestingly, additional rare homozygous variants of the *LTBP1*, *EMILIN1*, *MINAR1*, *DHS38*, and *MYO15A* genes were also identified in the siblings; however these genes were excluded from further analysis based on “inconsistently predicted” pathogenicity scores and/or their lack of association with neurodevelopmental abnormalities. These findings, detailed in Results, should be also summarized in the Abstract.

2) The human L126P β4 mutation was studied using “the corresponding rat β4 mutant L125P”. In the Abstract and main text, the authors should explain whether they refer to a heterologously expressed rat β4 clone – the current formulation is confusing. The paper should also clarify that the α-subunit in the study is a rabbit Cav2.1 channel clone (currently only the accession number is given). Accession number and/or source of the α_2_δ-1 subunit should be also included. The authors should also explain why α_2_δ-1 was used – the Abstract nominates α_2_δ-2 as the main component of the neurotransmitter release Cav2.1 channel complex.

3) Immunofluorescent labelling and IP was used to visualize Cav2.1 channel complexes in established cellular expression systems. Presence or absence of co-clustering, indicating Cav2.1/β4 interaction is convincingly demonstrated in a series of figures. The TNIK- β4 interaction suggested in Fig. 5 is an exciting result. However, the TNIK- β4 “complex formation” needs follow-up study and the statement referring to this should be softened in the abstract. It would be worthwhile discussing suitable experiments for investigating relevant aspects of synaptic physiology capable of revealing the impact of β4 dysfunction. Literature data indicate that mice carrying a knock-out mutation in TNIK have impaired postsynaptic and nuclear signalling pathways (Coba et al. 2012. J Neurosci. 32:13987–99).

4) Barium currents were studied in HEK cells heterologously co-expressing Cav2.1, α2δ-1, and wild-type or L125P β4 subunits (Fig. 7). Most of the investigated parameters of the current were unchanged, except the time course of inactivation (L125P β4 versus wild-type; Figs. 7E,F). Hence the statement that “current modulation of heterologously expressed channels remained intact” should be adjusted in the Abstract.

­*Minor*

1) The source of dysgenic myotubes (GLT line?) should be specified in the methods. Similarly, the source of hippocampal neurons (mice?) should be included. Currently only references are given.

2) In Fig. 2, higher magnification of some of the images and/or showing the corresponding phase-contrast images would be useful.

3) In Fig. 7B, the unit for the y-axis should be pA/pF. The legend for this figure should also indicate that α2δ-1 was included in the transfection mix.

4) I assume the authors use N and, respectively, n, to refer to numbers of independent experiments and the number of individual cells within the experiments. If so, this should be explained in the Materials and Methods. What was the N for Figs. 3 and 6?

5) The amount of co-transfected cDNA in heterologous expression experiments should be specified in all cases.

6) Data in Figs 7E,F suggest that it would be worthwhile investigating current availability of Cav2.1/L125P β4 versus Cav2.1/wild-type β4, especially the parameters of intermediate & slow inactivation and the use-dependence (during repetitive depolarizations). Although the absence of such data does not critically impact this study, the authors should include the parameters of voltage dependence of inactivation– if available.
