# Supplementary material for: A homozygous missense variant in CACNB4 encoding the auxiliary calcium channel beta4 subunit causes a severe neurodevelopmental disorder and impairs channel and non-channel functions
Source: PLoS Genet. 2020 Mar 16;16(3):e1008625. doi: 10.1371/journal.pgen.1008625 (PMC7176149; doi:10.1371/journal.pgen.1008625)
Supplement: Attachment — Submitted filename: CACNB4 Revision letter PLOS Genetics v4.docx [file pgen.1008625.s006.docx]

*PLOS Genetics*

Page 1/12

Editor-in-Chief

Gregory S. Barsh

Guest Editor

Ingo Helbig

December 10, 2019

**RE: PGENETICS-D-19-01072**

Dear Dr. Barsh,

dear Dr. Helbig,

Please find attached our revised manuscript entitled “A homozygous missense variant in *CACNB4* encoding the auxiliary calcium channel β_4_ subunit causes a severe neurodevelopmental disorder and impairs channel and non-channel functions”. We thank you and the four reviewers for valuable and constructive comments and suggestions.

A detailed list of our responses to your comments, the reviewer comments and a description of the changes made in the manuscript are provided below.

We hope that the revised version will prove acceptable for publication in your journal.

On behalf of all co-authors, we thank you for your assistance with our manuscript, and look forward to hearing from you.

Sincerely,

Bernhard E. Flucher, PhD	Kerstin Kutsche, PhD

***ANSWERS TO EDITORS’ AND REVIEWERS’ COMMENTS***

Editor and reviewer comments are italicized, responses in plain text. Changes in the manuscript and legends are marked in yellow.

***Editors’ comments to authors:***
*While the functional studies are thorough and well executed, a general question raised by the reviewers was whether the homozygous variants in CACNB4 was in fact causative in the family reported in your study, given that it represents a missense variant in a consanguineous population that is not well represented in population databases.

While it is often impossible to provide independent genetic evidence for ultra-rare genetic diseases as identifying further individuals with bi-allelic changes in this genes may be impossible, this uncertainty may be addressed in the revised version and you and your team may consider querying available genomic resources (DDD, epilepsy/autism datasets in dbgap) whether additional individuals with bi-allelic CACNB4 variants have been identified.*

We thank the Editors for their advice. As suggested, we searched available genomic resources and large whole-exome and whole-genome sequencing studies for additional individuals with biallelic *CACNB4* variants, but did not identify any. In addition, we uploaded *CACNB4* to GeneMatcher [1] and received the following matches:

1. Heterozygous deletion-insertion variant in *CACNB4* in an individual and one parent with myasthenia.
2. *De novo* missense variant in *CACNB4* in a patient with developmental delay and a diagnosis of autism.
3. *De novo* missense variant in *CACNB4* in a patient with developmental delay.

We added the information to the manuscript and also mentioned a degree of uncertainty that the biallelic missense variant in *CACNB4* might not be pathogenic (pages 8-9, lines 202-221).

**General reply to specific reviewers’ comments:**

Reviewers #3 and #4 asked for the biological consequences and possible pathomechanism underlying the p.(Leu126Pro) mutation in *CACNB4*. We aimed at addressing this point and searched the β_4_ literature to find possible pathways regulated by β_4_. β subunits have been shown to act in a voltage-gated calcium channel-independent manner in the cell [2, 3].

We and others have demonstrated nuclear translocation of the β_4_ subunit and regulation of gene transcription by β_4_ [4-6]. HEK293 cells have been used as model system to demonstrate differential expression of several genes upon ectopic expression of β_4_-EGFP compared to EGFP alone in this non-neuronal cell line. For example, the genes *NFKB2* and *SPATA17* were upregulated, while *DDX6* and *PTPRO* were downregulated in HEK293 cells overexpressing β_4_-EGFP [5]. To determine the effect of β_4b_ wildtype and the β_4b_-L125P mutant on transcriptional regulation, we isolated RNA from HEK293T cells transiently transfected with β_4b_-V5, β_4b_-L125P-V5 or empty vector followed by cDNA synthesis and quantitative reverse transcription PCR (RT-qPCR) using primers specific for the genes *NFKB2*, *SPATA17*, *DDX6* and *PTPRO*. Three independent RT-qPCR experiments revealed no significant difference in relative expression of the aforementioned genes in HEK293T cells transfected with β_4b_-V5, β_4b_-L125P-V5 or empty vector (data not shown). The data indicate that in our study expression of these genes was not regulated by heterologous expression of β_4b_ (wildtype or L125P).

We next turned our attention to a paper by Rima and colleagues (2017) who showed a β_4_-mediated inhibition of the Wnt/β-catenin signaling pathway in a genetically engineered human hepatocellular carcinoma cell line harboring a constitutive level of Wnt/β-catenin activity. Because expression of the wild-type β_4_ subunit induces inhibition of Wnt signaling by interaction with the transcription factor TCF4, the authors concluded that β_4_ acts as a repressor of TCF4. While the L125P mutation abolished binding of β_4_ to TCF4, probably due to loss of β_4_’s ability to be targeted to the nucleus [7], the β_4_-L125P mutant had no effect on the Wnt pathway. To further examine a possible role of β_4_ in Wnt signaling, we performed a β-catenin reporter assay in β_4b_ wildtype- and β_4_-L125P mutant-expressing HEK293T cells (Figure 1). We used a luciferase reporter construct encoding *Photinus*/Firefly luciferase under the control of a β-catenin-responsive promoter. According to the published data, we expected reduced firefly luciferase activity in Wnt3a-stimulated HEK293T cells expressing β_4_ wildtype (reflecting reduced Wnt signaling activity) in comparison to cells transfected with empty vector. Under basal conditions, expression of β_4b_-L125P mutant but not β_4b_ wildtype caused a slight increase in relative firefly luciferase activity compared to cells transfected with empty vector (Figure 1, right panel). Wnt3a stimulation of empty vector-transfected HEK293T cells resulted in higher firefly luciferase activity than in untreated cells indicating successful stimulation of the Wnt pathway. However, expression of both β_4b_ wildtype and -L125P mutant caused a similar reduction in relative firefly luciferase activity by 15-20 % compared to cells transfected with empty vector (Figure 1, left panel). Thus, this assay did not reveal any difference in inhibition of the Wnt signaling pathway in HEK293T cells expressing β_4b_ wildtype or the β_4b_-L125P mutant (Figure 1).

Figure 1

HEK293T cells were transiently transfected with a mixture of 8x TOPFlash luciferase reporter construct coding for *Photinus*/Firefly luciferase under control of a β-catenin-responsive promoter and a plasmid coding for *Renilla* luciferase driven by a CMV promoter. In addition, empty vector, β_4b_-V5 or β_4b_-L125P-V5 expression construct was co-transfected as indicated. Cells were starved in 1% FBS/DMEM overnight followed by incubation with 200 ng/mL Wnt3a for 4 h to stimulate Wnt signaling or left untreated. Afterwards, dual-luciferase reporter assay was performed according to manufacturers’ instructions (Promega, #1910). The mean of four independent experiments is given (n = 4). One-way ANOVA followed by Bonferroni *post-hoc* test for multiple comparisons was used for statistical analysis: * *p* ≤ 0.05; ** *p* ≤ 0.01; ns, not significant.

Finally, we monitored the amount of the Wnt signaling components β-catenin, GSK3β and TCF4 in Wnt3a-stimulated or unstimulated HEK293T cells expressing β_4b_ or β_4b_-L125P by immunoblotting. We did not observe any difference in the amount of β-catenin, GSK3β and TCF4 (Figure 2).

Figure 2

HEK293T cells were transfected with empty vector (EV), β_4b_-V5 or β_4b_-L125P-V5 expression construct as indicated. Next day, Wnt signaling was stimulated by incubation of cells with 100 ng/mL Wnt3a for 6 h. Subsequently, cells were lysed followed by immunoblotting using the indicated antibodies. V5-tagged β_4b_ proteins were detected by anti-V5-HRP antibody. Immunoblots of one experiment are shown.

Next, we examined pathways downstream of the β_4b_ target TNIK. We determined the phosphorylation status of the c-Jun N-terminal kinase (JNK) and its downstream target c-Jun, as Fu et al. (1999) reported that JNK can be phosphorylated by TNIK [8]. We hypothesized that the interaction between β_4b_ and TNIK might affect JNK signaling. Consistent with previous studies, JNK phosphorylation was increased in Wnt3a-treated cells in comparison to untreated cells (Figure 2). However, we observed no difference in the amount of phosphorylated JNK and c-Jun in HEK293T cells expressing β_4b_ or β_4b_-L125P (Figure 2).

In a previous study, overexpression of TNIK has been shown to result in disruption of F-actin structure [8]. To analyze if the abolished interaction between β_4b_-L125P and TNIK has a possible effect on the actin cytoskeleton, we transfected HeLa cells with β_4b_-V5, β_4b_-L125P-V5 or empty vector, stained F-actin by TexasRed-coupled Phalloidin and visualized cells expressing β_4b_ (wildtype or L125P mutant) by using an anti-V5 antibody and a fluorescent-dye conjugated secondary antibody. Again, we could not observe any difference in the amount and formation of F-actin-containing stress fibers in β_4b_ wildtype- or β_4b_ L125P mutant-expressing HeLa cells (Figure 3).

Figure 3

HeLa cells were transfected with empty vector, β_4b_-V5 or β_4b_-L125P-V5 expression construct as indicated. Next day, cells were fixed with PFA, blocked, and V5-tagged β_4_ proteins were stained by an anti-V5 antibody followed by AlexaFluor488-coupled anti-mouse antibody. F-actin was visualized by using TexasRed-coupled Phalloidin, and nuclear DNA was labeled by DAPI. Representative images of one experiment are shown. Scale bar, 20 µm.

The study by Gonzalez-Gutierrez et al. (2007) demonstrated that the auxiliary β subunits promote endocytosis through their Src homology 3 domain via dynamin interaction, thereby regulating the turnover of voltage-gated calcium channels and other proteins in the cell membrane [9]. Because the epidermal growth factor (EGF) is also internalized via dynamin-mediated endocytosis, we studied the effect of β_4b_ and β_4b_-L125P expression on EGF internalization. We incubated serum-starved, β_4b_ wildtype or β_4b_ L125P-expressing HeLa cells with ice-cold 1 µg/mL AlexaFluor555-coupled EGF in 0.3% BSA/DMEM for 1 h at 4°C. Internalization of EGF was induced by addition of pre-warmed 0.3% BSA/DMEM for 5, 10 or 15 min. Subsequently, remaining EGF at the cell surface was removed by extensive washing. V5-tagged β_4b_ subunits were visualized by immunocytochemistry using an anti-V5 antibody and an AlexaFluor488-coupled secondary antibody. The signal of AlexaFluor555-coupled EGF increased over time, with increasing exposure time of pre-warmed medium, independent of the transfected construct (β_4b_-V5, β_4b_-L125P-V5 or empty vector) (Figure 4). Together, no significant difference in the distribution and amount of internalized EGF was detected in HeLa cells expressing β_4b_ -V5 or β_4b_-L125P-V5 at the analyzed time points.

Figure 4

HeLa cells were transfected with β_4b_-V5, β_4b_-L125P-V5 or empty vector. Cells were starved in 0.3% BSA/DMEM for 2 h followed by incubation with ice-cold 1 µg/mL AlexaFluor555-coupled EGF in 0.3% BSA/DMEM for 1 h at 4°C. Internalization of EGF was allowed for 5, 10 or 15 min by treatment of cells with pre-warmed 0.3% BSA/DMEM. Subsequently, cell surface EGF was removed by intensive washing with ice-cold acidic wash puffer (0.2 mM acetic acid, 0.5 mM NaCl, pH 2.5), ice-cold 0.3% BSA/DMEM twice and PBS on ice once. Cells were fixed with 4% PFA, blocked, and β_4b_-V5 and β_4b_-L125P-V5 were visualized by an anti-V5 antibody and a secondary AlexaFluor488-coupled anti-mouse antibody. Nuclear DNA was labeled by DAPI. Representative images of one experiment are shown. Scale bar, 20 µm.

Taken together, by analyzing different pathways in HEK293T cells transiently expressing β_4b_ wildtype or β_4b_-L125P mutant we were not able to detect significant differences in the selected biological read-outs. These data may point to specific effects of the β_4b_-L125P mutant in neuronal (as shown by our data presented in figures 3 and 4) versus non-neuronal cells and indicate the need of more extensive and time-consuming studies in primary hippocampal neurons and/or rodent models to define the pathomechanism of the *CACNB4* variant p.(Leu126Pro) in the future.

*Comments to the Authors:*

*Reviewer #1: The manuscript by Coste de Bagneaux and colleagues examines the functional impacts of a homozygous missense mutation in the CACNB4 gene encoding the β4 subunit. Patients - two siblings of first-degree consanguineous healthy parents, carrying the L126P β4 mutation exhibit severe neurodevelopmental disorder, including intellectual disability and other comorbidities. The unique clinical data directly motivate the study. The results suggest that the mutation impairs presynaptic Cav2.1 channel function and disrupts nuclear targeting of β4. The paper is well-written and, in most cases, the conclusions are justified by results. Nuclear targeting of β4 is convincingly demonstrated. However, the suggested TNIK-β4 complex formation should be softened in the abstract.*

We agree and softened the statement in the abstract (page 2, line 58).

*In neurons, the auxiliary β4 subunit is associated with the pore-forming Cav2.1 (α-) subunit and modulates neurotransmission; β4 can also function as a chaperon, facilitating Cav2.1 channel complex formation and regulating surface expression. Unlike other β subunits, β4 can specifically be targeted to the nuclei of neurons to partner proteins involved in transcription and cell proliferation. At the molecular level, β4 interacts with the AID domain of the α-subunit, which binds to a hydrophobic groove of the β4’s guanylate kinase (GK) domain. Mutations in the pore-forming Cav2.1 or the auxiliary subunits are often associated with neurological disease; specifically, mutations in β4 can be implicated in epilepsy and ataxia in humans. The L126P residue-change in the GK domain likely impairs the intramolecular interaction of GK with the tandem srk homology (SH3) module of β4.*

***Major comments***

*1) The in silico pathogenicity prediction methods provided high scores for the L126P β4 and suggested this mutation was responsible for the neurological disease in the patients. Interestingly, additional rare homozygous variants of the LTBP1, EMILIN1, MINAR1, DHS38, and MYO15A genes were also identified in the siblings; however these genes were excluded from further analysis based on “inconsistently predicted” pathogenicity scores and/or their lack of association with neurodevelopmental abnormalities. These findings, detailed in Results, should be also summarized in the Abstract.*

We updated the section in the manuscript discussing the rare homozygous variants in a total of six genes, including *CACNB4*, in the two siblings. *LTBP1* has been reported as disease gene candidate for primary platelet secretion defects (page 7, lines 162-163) and *EMILIN1* as possible new disease gene for an autosomal-dominant connective tissue disorder (page 7, lines 165-167). Furthermore, we discussed the constraint score for all six genes and used MetaDome, another *in silico* tool which provides profiles of genetic tolerance through aggregation of homologous human protein domains. Thus, by a combination of *in silico* tools, animal model, clinical and genetic data, we suggest the homozygous p.(Leu126Pro) amino acid substitution in *CACNB4* to underlie the neurological disorder in the two siblings (page 9, lines 210-221).

As suggested by the reviewer, we included the finding of six rare homozygous variants in the two siblings in the abstract (page 2, lines 41-45).

*2) The human L126P β4 mutation was studied using “the corresponding rat β4 mutant L125P”. In the Abstract and main text, the authors should explain whether they refer to a heterologously expressed rat β4 clone – the current formulation is confusing. The paper should also clarify that the α-subunit in the study is a rabbit Cav2.1 channel clone (currently only the accession number is given). Accession number and/or source of the α_2_δ-1 subunit should be also included. The authors should also explain why α_2_δ-1 was used – the Abstract nominates α_2_δ-2 as the main component of the neurotransmitter release Cav2.1 channel complex.*

We changed the wording in the abstract accordingly (page 2, lines 46-47).

The methods have been updated to include all species names and Genbank accession numbers (page 22, lines 576-578). As outlined in the abstract, α_2_δ-2 is the “most-likely” partner of endogenous Cav2.1 channels, although an ultimate proof is still missing. However, upon heterologous expression and even in cultured neurons distinct α_2_δ subunit isoforms have similar effects on current densities and expression of heterologously expressed α_1_ subunits (e.g. [10, 11]). Thus, to facilitate comparison with previous studies we used the rabbit α_2_δ-1 isoform for co-expression studies.

*3) Immunofluorescent labelling and IP was used to visualize Cav2.1 channel complexes in established cellular expression systems. Presence or absence of co-clustering, indicating Cav2.1/β4 interaction is convincingly demonstrated in a series of figures. The TNIK- β4 interaction suggested in Fig. 5 is an exciting result. However, the TNIK- β4 “complex formation” needs follow-up study and the statement referring to this should be softened in the abstract. It would be worthwhile discussing suitable experiments for investigating relevant aspects of synaptic physiology capable of revealing the impact of β4 dysfunction. Literature data indicate that mice carrying a knock-out mutation in TNIK have impaired postsynaptic and nuclear signalling pathways (Coba et al. 2012. J Neurosci. 32:13987–99).*

We thank the reviewer for the suggestion and modified the Discussion section accordingly (page 21, lines 531-538).

*4) Barium currents were studied in HEK cells heterologously co-expressing Cav2.1, α2δ-1, and wild-type or L125P β4 subunits (Fig. 7). Most of the investigated parameters of the current were unchanged, except the time course of inactivation (L125P β4 versus wild-type; Figs. 7E,F). Hence the statement that “current modulation of heterologously expressed channels remained intact” should be adjusted in the Abstract.*

As suggested by the reviewer, we specified the current properties that were still modulated by β_4b_-L125P expressed in heterologous cells (page 2, lines 53-54).

*­Minor*

*1) The source of dysgenic myotubes (GLT line?) should be specified in the methods. Similarly, the source of hippocampal neurons (mice?) should be included. Currently only references are given.*

The GLT cell line has been generated by us. Therefore, the reference (Powell et al. 1996) correctly indicates the source (page 23, line 588).

The source of hippocampal neurons is given (page 23, lines 592-593).

*2) In Fig. 2, higher magnification of some of the images and/or showing the corresponding phase-contrast images would be useful.*

Inserts with magnified regions of interest have been added to the images in Figure 2 as suggested.

*3) In Fig. 7B, the unit for the y-axis should be pA/pF. The legend for this figure should also indicate that α2δ-1 was included in the transfection mix.*

Corrected as suggested (page 43, lines 1136-1137).

*4) I assume the authors use N and, respectively, n, to refer to numbers of independent experiments and the number of individual cells within the experiments. If so, this should be explained in the Materials and Methods. What was the N for Figs. 3 and 6?*

Yes, “N” refers to the number of independent experiments and “n” to the number of individual cells analyzed. We included a sentence in the Material and methods section (page 26, lines 681-682).

We included the following sentence in the legend of figure 3: “Representative images of three independent experiments are shown (see Results for statistics).”

We included the following sentence in the legend of figure 6: “Representative images of two independent experiments are shown.”

*5) The amount of co-transfected cDNA in heterologous expression experiments should be specified in all cases.*

We added the following sentence in the Material and Methods section: “HEK293T cells were transiently transfected with 1 µg (immunocytochemistry) or 5 µg of plasmid DNA (Co-immunoprecipitation) using TurboFect (Thermo Fisher Scientific) for 6 h according to the protocol provided and cultured in DMEM overnight.“ (page 23, lines 607-610).

*6) Data in Figs 7E,F suggest that it would be worthwhile investigating current availability of Cav2.1/L125P β4 versus Cav2.1/wild-type β4, especially the parameters of intermediate & slow inactivation and the use-dependence (during repetitive depolarizations). Although the absence of such data does not critically impact this study, the authors should include the parameters of voltage dependence of inactivation– if available.*

Because β_4b_-L125P still promoted membrane insertion and modulation of activation properties in tsA201 cells, while complex formation in differentiated nerve and muscle cells failed, we did not analyse further electrophysiological parameters like current availability or the voltage-dependence of inactivation in the heterologous cell system. Whatever differences we might determine may not be indicative of the situation in native cells.

*Reviewer #2: The authors provide a very nice article which solves a long debate about the role of variants in CACNB4 to cause disorders of the nervous system in humans. Although they only report about one family with two recessively affected sibs, their functional work is highly comprehensive and demonstrates the pathological role of the homozygous variant undoutedly. Interestingly, a simple expression in HEK or tsA201 cells of the mutant vs. WT subunit together with alpha1 and alpha2delta subunits would not have revealed this pathological role. But the extensive studies in neurons, myotubes and other cells including biochemistry and immunohistochemistry reveal the defective interaction of the mutant CACNB4 subunit with various other proteins disrupting both the electrophysiological function of the P/Q- or L-type Ca channel complex and the interaction with nuclear targeting and signaliing. These reeults are highly convincing and well performed. I have no critique or suggestions for changes.*

*The study is highly important, since it terminates a long debate about eventually disease-causing heterozygous variants which becomes very unlikely due to their study (although a smaller contribution of disease-modifying effects of het variants can still not - may be never - be excluded).*

We thank the reviewer for his positive feedback.

*Reviewer #3: In this manuscript the authors describe a rare homozygyous variant in CACNB4 that is found in 2 siblings with a severe neurodevelopmental disorder. The clinical, genetic and functional data are of a high standard. The manuscript is generally well written although it could be tightened up a little—especially the discussion but also bits of the results. The authors provide strong evidence that the CACNB4 disrupts some physiological processes. However, I am not completely convinced that the leu126Pro is the cause of the severe neurodevelopmental phenotype seen in the siblings.*

*First, there are 6 candidate variants that could be responsible for disease. The authors systematically rule out those other than CACNB4 based on a combination of in silico, animal model and clinical data. The case for exclusion is not that strong in my opinion with each of the ‘exclusion methods’ frequently shown to be wanting. For example, in silico analysis is particularly fraught. While I accept that completing functional analysis on all is not practical it remains the best way of ruling out these variants (although even this approach has its issues).*

We thank the reviewer for the comment.

In the era of whole-exome and whole-genome sequencing human geneticists are tackling with many sequence variants. Particularly in consanguineous families we usually find several rare and private homozygous variants in the affected child/siblings. Assigning pathogenicity to any of the rare variants can be challenging. We are aware of the difficulty to find the causative variant among several homozygous variants. However, *in silico* pathogenicity prediction tools that have improved over time facilitate assigning possible pathogenicity to a rare variant. Nevertheless, functional studies are mandatory to provide evidence for pathogenicity of a selected variant. Indeed, functional analysis on all six candidate variants is preferable but not feasible. We updated the section in the manuscript discussing the rare homozygous variants in a total of six genes, including *CACNB4*, in the two siblings. *LTBP1* has been reported as disease gene candidate for primary platelet secretion defects (page 7, lines 162-163) and *EMILIN1* as possible new disease gene for an autosomal dominant connective tissue disorder (page 7, lines 165-167). Furthermore, we discussed the constraint score for all six genes and used MetaDome, another *in silico* tool which provides profiles of genetic tolerance through aggregation of homologous human protein domains. Although we are still convinced of the pathogenic nature of the *CACNB4* variant, we now acknowledge a degree of uncertainty that the homozygous missense variant in *CACNB4* might not be pathogenic (page 9, lines 210-221).

*The functional impact of the leu126Pro variant goes someway in arguing that it is potentially pathogenic. However, all the assays that showed differences relate to binding and/or trafficking. The biological consequences of these is not clear to me. It would be good to see a tractable output that had a biological relevance and point to a pathomechanism. The only assay the specifically looks at a functional output that is tractable is the electrophysiology in which the variant has no impact.*

In a first attempt to address this important issue we examined several pathways suggested to be directly or indirectly regulated by β_4_ (please see “**General reply to specific reviewers’ comments”** on the first page). Unfortunately, these pilot experiments performed in HEK293T cells transfected with β_4b_ wildtype and L125P mutant did not indicate an effect of L215P in any of these cases. Evidently, functional consequences need to be studied in neuronal cell systems or in the intact organism, which goes beyond of what could be done in the course of the revision.

*Perhaps the most compelling way to confirm pathogenicity is the engineering of a rodent model. With CRISPR technology this is no longer prohibitive.*

We agree with the reviewer that a knock-in mouse model would be the ultimate test to determine the *in vivo* biological consequences of the *CACNB4* p.(Leu126Pro) variant. Obviously, this is beyond the scope of the current manuscript.

*In summary, although the manuscript contains very solid data it lacks compelling evidence that the studied CACNB4 leu126Pro variant is causing disease.*

We respectfully disagree with the reviewer. By biochemistry and immunohistochemistry in neurons, myotubes and other cells we show that the p.(Leu126Pro) variant disturbs various biological functions of the β_4b_ subunit and likely underlies the phenotype in the two siblings.

*Reviewer #4: In this study, de Bagneaux and colleagues report a homozygous mutation in CACNB4 identified in two siblings with severe intellectual disability, blindness, epilepsy, movement disorder and cerebellar atrophy. They report functional analysis of the identified variant using the expression in cultured hippocampal neurons, tSA cells and cultured myotubes and suggest that the mutant beta 4 subunit shows reduced incorporation in the presynaptic calcium complexes, absence of nuclear targeting and abolished interaction with a neuronal kinase. However, coexpression of the mutant with the Cav2.1 in tsA201 cells affected the calcium currents in a similar way as the beta 4 wild type. The authors suggest that their data provide evidence for the pathogenicity of the detected variant and corroborate the role of CACNB4 in human disease.*

*This is a well written and structured manuscript providing elaborate analysis and some interesting insights into the function of the beta 4 subunit and its potential role in the severe neurodevelopmental disorders. The major concern in my view is that while L125P variant seems to affect localization and interactions of the beta 4 subunit with other proteins, there is no clear indication in this manuscript how this may lead to the severe disease seen in the two patients.*

*This question can be addressed in several ways that could corroborate the conclusions of the study. Firstly, since the variant is present in unaffected parents, a comparison to the heterozygous expression would be a very useful control.*

To address this interesting point we analyzed formation of β_4b_-Ca_V_1 channel complexes and β_4b_ nuclear targeting in the muscle expression system by coexpressing equal amounts of wild-type β_4b_-GFP and β_4b_-L125P-V5 and double-labeling with anti-GFP and anti-V5 antibody, respectively. As expected wild-type β_4b_-GFP was incorporated in channel complexes (clusters), while β_4b_-L125P-V5 remained diffusely distributed in the cytoplasm. In addition, in cells that showed β_4b_-GFP nuclear targeting, mutant β_4b_-L125P-V5 remained excluded from the nuclei (S1 Fig). These results demonstrate that in the presence of mutant β_4b_-L125P the wild-type β_4b_ subunit functions normally. This observation is consistent with the fact that heterozygous parents of the two affected siblings were healthy (pages 10-11, lines 258-266).

*Furthermore, given the discrepancy between expression in heterologous systems and neurons, a functional assay pointing at the altered neuronal/synaptic activity would be indicative of the underlying disease mechanism.*

The reviewer refers to an apparent discrepancy between heterologous and homologous expression systems. Actually, it has previously been demonstrated that modulation of heterologously expressed channels depends on the availability and concentration of beta subunits rather than on a tight physical interaction [12, 13]. Accordingly, we do not agree that the observations in heterologous expression systems contradict the observations in neurons. However, we agree with the reviewer that altered neuronal/synaptic activity could provide additional insights into pathogenicity. Studying the consequences of overexpression in neurons depends on the competition of overexpressed β_4b_ with endogenously and abundantly available beta subunits. As β_4b_-L125P is not trafficked into the distal axon and thus not incorporated into presynaptic terminals, competition with endogenous synaptic beta subunits is not to be expected and hence such experiments will remain inconclusive.

*Lastly, any insights into the changes of the gene expression resulting from the altered nuclear translocation or interaction with TNIK would be useful to understand the proposed effect of the variant.*

Please see “**General reply to specific reviewers’ comments”** on the first page for information on pilot experiments performed to address this point.

*The paper would also be improved by providing more consistent quantification of morphological and biochemical data. For instance, in Fig 3, have the authors considered neurite tracing and quantification? Also, axon hillock expression (Fig 4) should be quantified.*

We agree with the reviewer and have performed quantitative analysis of the expression level of normal and mutant β_4b_ in the distal axon and the axon hillock. The data are now part of the results section (page 11, lines 277-279 and page 13, lines 317-321), and the methods section has been updated accordingly (pages 25-26, lines 660-669).

*Fig 5 shows that the input lysates in both experiments had less mutant protein to start with. Is the reduced expression of the L126P part of the mechanism?*

We agree with the reviewer that heterologous expression of the β_4b_-L125P mutant seemed to be lower than β_4b_ wildtype expression in HEK293T cells. However, based on the overexpression system we cannot claim that the β_4b_-L125P mutant shows decreased stability and/or enhanced degradation.

**References:**

1.	Sobreira N, Schiettecatte F, Valle D, Hamosh A. GeneMatcher: a matching tool for connecting investigators with an interest in the same gene. Hum Mutat. 2015;36(10):928-30. pmid: 26220891; PubMed Central PMCID: PMCPMC4833888.

2.	Dolphin AC. Calcium channel diversity: multiple roles of calcium channel subunits. Curr Opin Neurobiol. 2009;19(3):237-44. pmid: 19559597.

3.	Campiglio M, Flucher BE. The role of auxiliary subunits for the functional diversity of voltage-gated calcium channels. J Cell Physiol. 2015;230(9):2019-31. pmid: 25820299; PubMed Central PMCID: PMCPMC4672716.

4.	Tadmouri A, Kiyonaka S, Barbado M, Rousset M, Fablet K, Sawamura S, et al. Cacnb4 directly couples electrical activity to gene expression, a process defective in juvenile epilepsy. EMBO J. 2012;31(18):3730-44. pmid: 22892567; PubMed Central PMCID: PMCPMC3442274.

5.	Ronjat M, Kiyonaka S, Barbado M, De Waard M, Mori Y. Nuclear life of the voltage-gated Cacnb4 subunit and its role in gene transcription regulation. Channels. 2013;7(2):119-25. pmid: 23511121; PubMed Central PMCID: PMCPMC3667881.

6.	Etemad S, Obermair GJ, Bindreither D, Benedetti A, Stanika R, Di Biase V, et al. Differential neuronal targeting of a new and two known calcium channel β4 subunit splice variants correlates with their regulation of gene expression. J Neurosci. 2014;34(4):1446-61. pmid: 24453333; PubMed Central PMCID: PMCPMC3898300.

7.	Rima M, Daghsni M, Lopez A, Fajloun Z, Lefrancois L, Dunach M, et al. Down-regulation of the Wnt/β-catenin signaling pathway by Cacnb4. Mol Biol Cell. 2017;28(25):3699-708.

8.	Fu CA, Shen M, Huang BCB, Lasaga J, Payan DG, Luo Y. TNIK, a Novel Member of the Germinal Center Kinase Family That Activates the c-Jun N-terminal Kinase Pathway and Regulates the Cytoskeleton. J Biol Chem. 1999;274(43):30729–37.

9.	Gonzalez-Gutierrez G, Miranda-Laferte E, Neely A, Hidalgo P. The Src Homology 3 Domain of the β-Subunit of Voltage-gated Calcium Channels Promotes Endocytosis via Dynamin Interaction. J Biol Chem. 2007;282(4):2156–62.

10.	Geisler S, Schopf CL, Obermair GJ. Emerging evidence for specific neuronal functions of auxiliary calcium channel alpha(2)delta subunits. Gen Physiol Biophys. 2015;34(2):105-18. pmid: 25504062; PubMed Central PMCID: PMCPMC4487825.

11.	Hoppa MB, Lana B, Margas W, Dolphin AC, Ryan TA. alpha2delta expression sets presynaptic calcium channel abundance and release probability. Nature. 2012;486(7401):122-5. pmid: 22678293; PubMed Central PMCID: PMCPMC3376018.

12.	Butcher AJ, Leroy J, Richards MW, Pratt WS, Dolphin AC. The importance of occupancy rather than affinity of CaV(beta) subunits for the calcium channel I-II linker in relation to calcium channel function. J Physiol. 2006;574(Pt 2):387-98. pmid: 16627564; PubMed Central PMCID: PMCPMC1817768.

13.	Gerster U, Neuhuber B, Groschner K, Striessnig J, Flucher BE. Current modulation and membrane targeting of the calcium channel alpha1C subunit are independent functions of the beta subunit. J Physiol. 1999;517 ( Pt 2):353-68. pmid: 10332087; PubMed Central PMCID: PMCPMC2269342.
